# Identification of volasertib-resistant mechanism and evaluation of combination effects with volasertib and other agents on acute myeloid leukemia

**DOI:** 10.18632/oncotarget.19632

**Published:** 2017-07-26

**Authors:** Yoshiya Adachi, Yuichi Ishikawa, Hitoshi Kiyoi

**Affiliations:** ^1^ Department of Hematology and Oncology, Nagoya University Graduate School of Medicine, Nagoya, Aichi, Japan

**Keywords:** AML, PLK1 inhibitor, volasertib, resistance, MDR1

## Abstract

Volasertib, a selective PLK1 inhibitor, was effective for acute myeloid leukemia (AML) patients in clinical trials. However, its efficacy was limited in mono-therapy, and a higher incidence of fatal events was revealed in the combination with low-dose cytarabine. Thus, optimization of combination therapy with volasertib and other agents is necessary for its clinical development, and the predictive factors for response or resistance to volasertib remain largely unknown. In this study, we investigated the resistance mechanism in volasertib-resistant cell lines and the combination effects with other agents, such as azacitidine (AZA), on AML cells. We identified that mutations in the ATP-binding domain of PLK1 and expression of MDR1 conferred resistance to volasertib. In the combination therapy, the effects of AZA differed among cells, but were prominent in the cells with higher GI_50_ values of volasertib in mono-therapy. Furthermore, we identified that the cells in G2/M phase were more sensitive to volasertib, and the PI3K/AKT pathway was up-regulated upon administration of volasertib. Combination therapies with the agents that caused cell cycle accumulation in G2/M phase or with PI3K inhibitor were highly potent against AML cells. Our findings provide strategies for further clinical development of volasertib and PLK inhibitors for AML.

## INTRODUCTION

The polo-like kinase (PLK) family are serine/threonine protein kinases and consist of five members: PLK1, PLK2, PLK3, PLK4 and PLK5 [1–3]. These PLKs regulate many steps of the cell cycle, centriole duplication, DNA replication, centrosome separation, maturation, mitotic entry, spindle formation, chromosome segregation and cytokinesis. Among the five members of the PLK family, PLK1 plays a crucial role in cell mitosis and is highly expressed during the G2/M and S phase [4, 5]. As PLK1 is overexpressed in several cancer cells, it has been recognized as an attractive target for cancer therapy and many PLK inhibitors are under investigation [6, 7]. Volasertib is an ATP-competitive PLK inhibitor and potently inhibits PLK1, PLK2 and PLK3 with IC_50_ values of 0.87 nM, 5 nM and 56 nM, respectively [8]. Additionally, it does not exhibit any inhibitory effects against a panel of more than 50 other kinases at concentrations up to 10 μM. Previous studies demonstrated that volasertib inhibited cell proliferation in a variety of cancer cell lines *in vitro* and its efficacy was also confirmed in tumor xenograft mice models with solid tumors and acute myeloid leukemia (AML) [6, 9, 10]. Moreover, volasertib was clinically efficient in several cancers and prominent in the clinical trials against AML patients [11].

In clinical trials, volasertib mono-therapy was effective in a part of AML patients; however, its efficiency was limited and combination therapy with other anti-leukemia agents was considered [12]. The combination therapy of volasertib and low-dose cytarabine (LDAC) showed a superior response rate and overall survival than LDAC mono-therapy in AML patients who were not suitable for standard induction therapy [13]. However, a subsequently conducted phase III study (NCT01721876) could not demonstrate higher overall survival rates in the volasertib and LDAC treated patients than in those with LDAC mono-therapy [14]. Although volasertib and LDAC treated patients showed higher response rates, the higher incidence of fatal infection was observed. Therefore, optimization of the combination therapy with other agents, administration dosage and schedule was necessary for the clinical development. Combination therapy with azacitidine (AZA) or decitabine, was investigated in patients with myelodysplastic syndrome (MDS) (NCT01957644). However, the combination effects of volasertib and AZA are not well established in MDS and AML cells. Moreover, it is also required to identify the predictive factors for its clinical response and the resistance of volasertib in AML cells.

In this study, we evaluated the efficacies of volasertib in mono- and combination-therapy with AZA or other agents against AML cell lines and primary AML cells. In addition, we established volasertib-resistant AML cell lines and demonstrated the resistant mechanism of volasertib in these cells. We also provide strategies to improve clinical outcomes for combination therapy with other agents.

## RESULTS

### The efficacy of volasertib in leukemia/lymphoma cell lines

**Figure 1:**
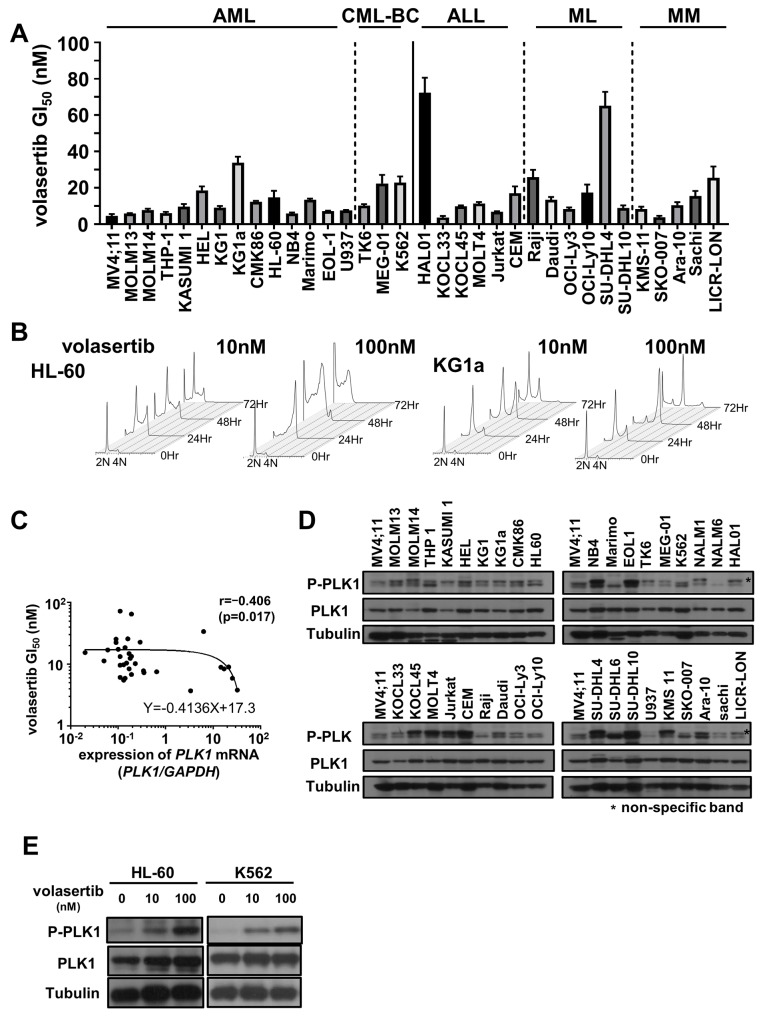
The efficacy of volasertib in a series of human hematological malignant cell lines **(A)** The GI_50_ values of volasertib in acute myeloid leukemia (AML), chronic myeloid leukemia in blast crisis (CML-BC), acute lymphoblastic leukemia (ALL), malignant lymphoma (ML) and multiple myeloma (MM) cell lines. Error bars represent the mean ± S.D. of at least three independent experiments. **(B)** Cell cycle analysis in AML cell lines after volasertib treatment. **(C)** The correlation between the GI_50_ values of volasertib and the expression levels of PLK1 mRNA in each cell line. The correlation coefficient was determined by the Spearman’s rank correlation coefficient. **(D)** The protein levels of PLK1 and phosphorylated PLK1 were determined by western blot analysis in cell line. **(E)** The protein expression of PLK1 and its phosphorylation levels at 24 hours after volasertib treatment in HL-60 and K562 cells.

To examine the efficacy of volasertib, we evaluated GI_50_ values of volasertib in a variety of human AML, chronic myeloid leukemia in blast crisis (CML-BC), acute lymphoblastic leukemia (ALL), malignant lymphoma (ML) and multiple myeloma (MM) cell lines. Volasertib was highly potent against most cell lines in mono-therapy (Figure 1A). Cell cycle analyses in HL-60 and KG1a cells resulted in an accumulation of cells with 4N DNA content, and subsequently the subG1 components were increased (Figure 1B and Supplementary Figure 1A).

To determine the biomarkers that predict the efficacy of volasertib in these cell lines, we examined the expression levels of *PLK1, PLK2* and *PLK3* mRNA, and the phosphorylation level of PLK1 before the volasertib treatment. *PLK1* mRNA was expressed in all cell lines, and there was a weak negative correlation between the GI_50_ value and the expression level of *PLK1* mRNA (Figure 1C and Supplementary Figure 1B). On the other hand, the expression levels of *PLK2* and *PLK*3 mRNA were quite lower than those of *PLK1* mRNA in the AML cell lines (Supplementary Figure 1C) and there were no significant correlations between the *PLK2* and *PLK3* mRNA expression levels, and the resistance to volasertib. Additionally, the PLK1 phosphorylation levels at the steady state were not associated with the efficacy of volasertib in cell lines (Figure 1D). We also examined the protein expression levels and phosphorylation levels of PLK1 after volasertib treatment by western blot. The phosphorylation levels of PLK1 was increased after volasertib treatment in a dose-dependent manner (Figure 1E).

### Establishment of volasertib-resistant cell lines

**Figure 2:**
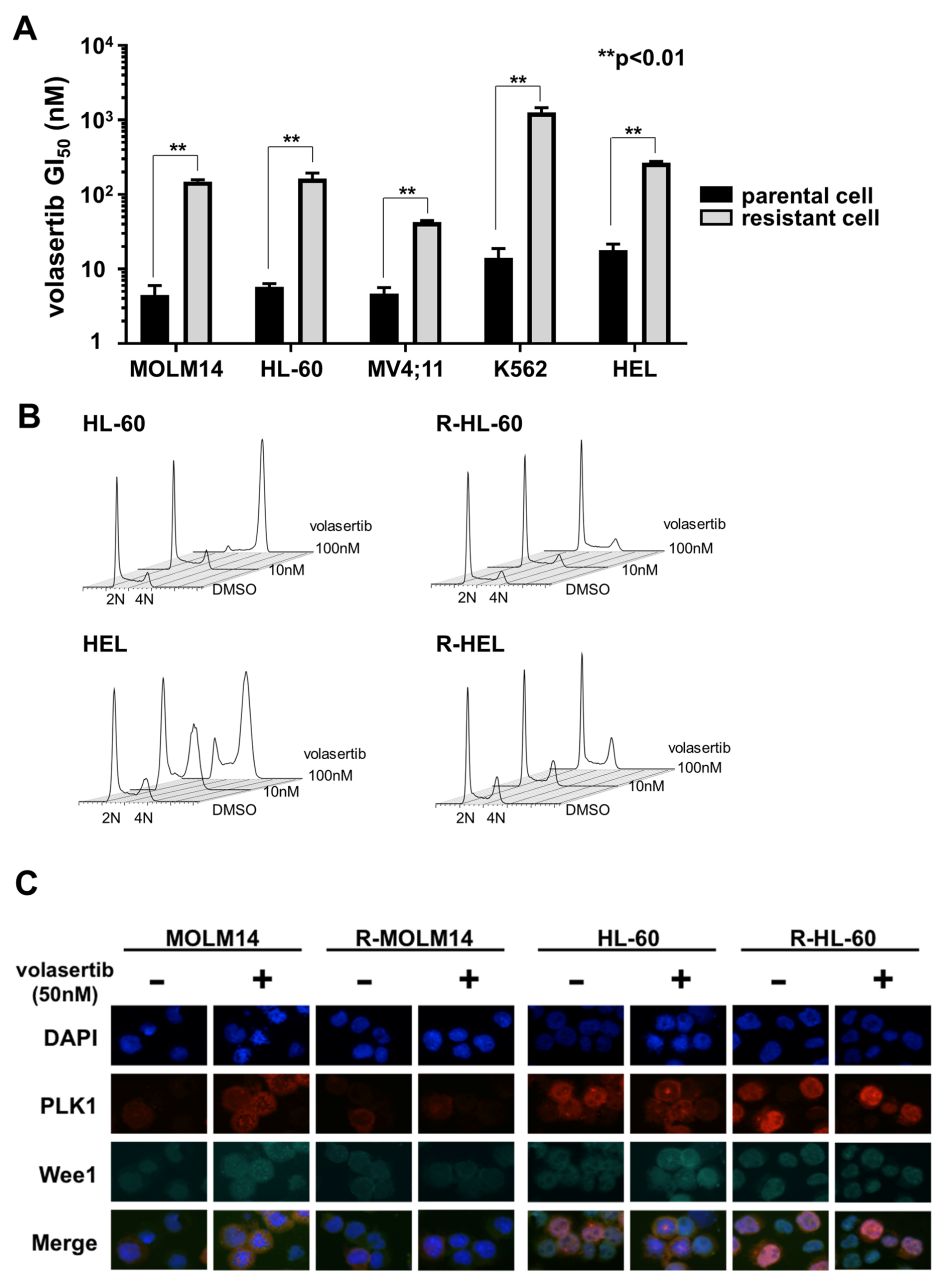
Establishment of volasertib-resistant cells and resistant mechanism of volasertib **(A)** The GI_50_ values of volasertib in both the parental and the volasertib-resistant cells. Error bars represent the mean values ± S.D. of at least three independent experiments. **(B)** The cell cycle analysis in both the parental and the volasertib-resistant cells after volasertib administration. **(C)** The protein expressions of PLK1 and Wee1 were evaluated by immunofluorescent staining. Both parental and volasertib-resistant MOLM14 and HL-60 cells were treated with 50 nM volasertib for 18 hours.

To explore the volasertib-resistant mechanism, we established volasertib-resistant MOLM14, HL-60, MV4;11, K562 and HEL (R-MOLM14, R-HL-60, R-MV4;11, R-K562 and R-HEL) cells. The half-maximal growth inhibitory concentration (GI_50_) values of volasertib against the parental and volasertib-resistant cells were 4.6 nM and 149.8 nM in MOLM14, 5.8 nM and 164.0 nM in HL-60, 4.6 nM and 42.8 nM in MV4;11, 14.1 nM and 1265.8 nM in K562, and 17.7 nM and 277.7 nM in HEL, respectively (Figure 2A). We examined the cell cycle status in both parental and volasertib-resistant cells at 24 hours after volasertib treatment (Figure 2B). Although G2/M arrest was observed in the parental cells dose-dependently, it was not observed in the volasertib-resistant cells. Next, we examined protein expression levels of Wee1, which is a substrate of PLK1 that is degraded after its phosphorylation, after volasertib administration by immunofluorescence staining and western blot (Figure 2C and Supplementary Figure 2). In parental cells, the expression of Wee1 was increased by volasertib administration, whereas it was very low in the volasertib-resistant cells.

### Mutations in the PLK1 ATP-binding domain conferred resistance to volasertib

**Figure 3:**
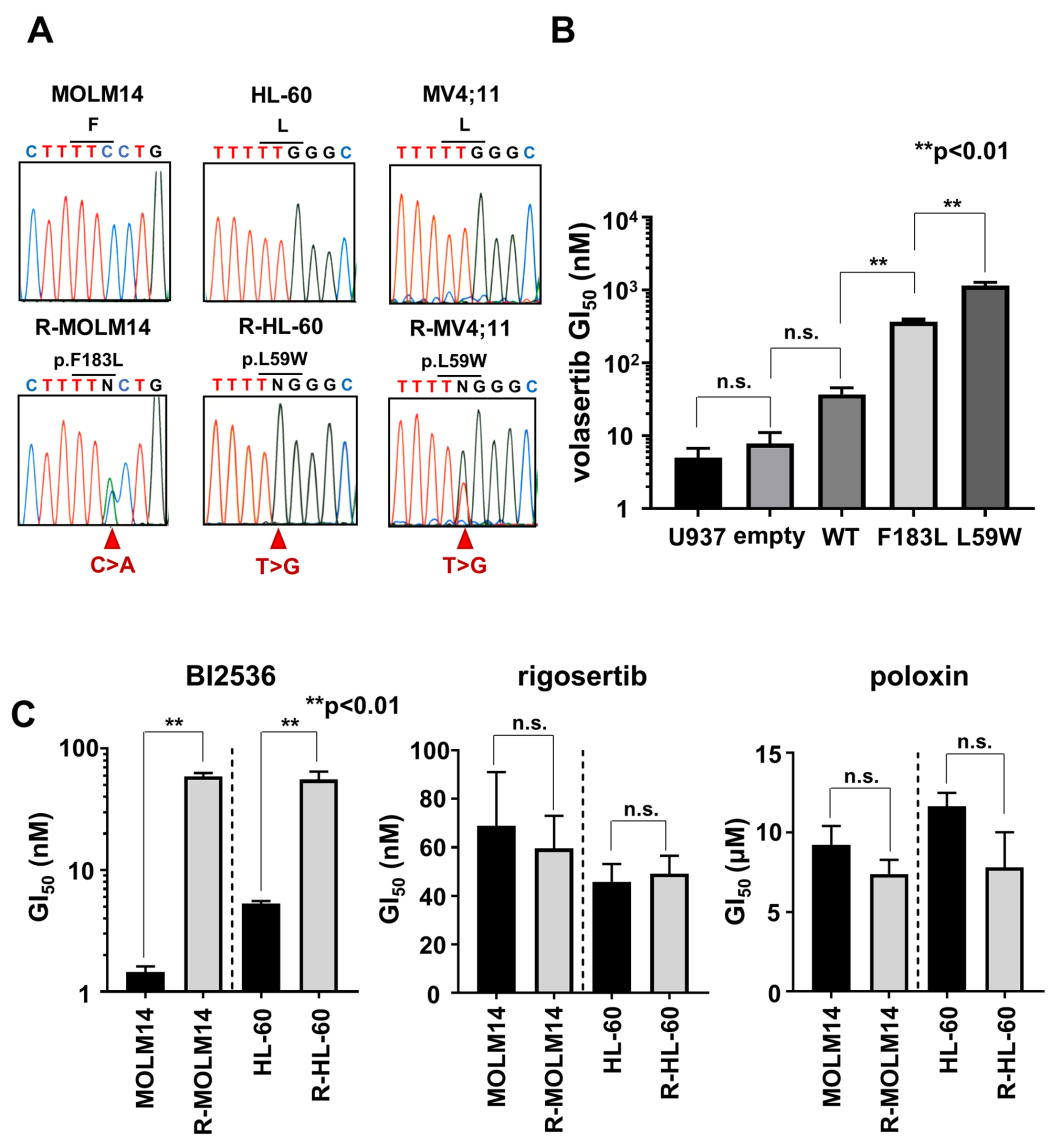
The mutations in *PLK1* ATP-binding domain conferred resistance to volasertib **(A)** Genomic DNA sequence analyses of *PLK1* in both the parental and the volasertib-resistant cells. **(B)** The GI_50_ values of volasertib on mutant *PLK1* transduced U937 cells. One-way ANOVA was performed. **(C)** The GI_50_ values of BI2536, rigosertib, and poloxin were determined in both parental and volasertib-resistant R-MOLM14 and R-HL-60. Error bars represent the mean values ± S.D. of at least three independent experiments.

We examined the resistant mechanisms of volasertib in these volasertib-resistant cells. We identified novel missense *PLK1* mutations in R-MOLM14 (p.F183L), R-HL-60 (p.L59W) and R-MV4;11 (p.L59W) (Figure 3A). These mutated residues are located in the ATP-binding domain of PLK1, which is key to the combining of volasertib with PLK1 (Supplementary Figure 3A). To confirm the impact of these mutations on volasertib sensitivity, mutant PLK1s were transduced into U937 cells (Supplementary Figure 3B). These mutant PLK1-expressing cells demonstrated resistance to volasertib; the GI_50_ values of volasertib in the wild type, F183L and L59W transduced cells were 37.1nM, 363.6 nM and 1150.9nM, respectively (Figure 3B). Consequently, these results indicated that mutations in ATP-binding residues conferred volasertib-resistance to leukemia cells. Next, we explored the efficacies of other PLK1 inhibitors in these volasertib-resistant cells. Although these volasertib-resistant cells were also resistant to BI2536, an ATP competitive PLK1 inhibitor [15], they were still sensitive to rigosertib [16], a substrate competitive inhibitor, and poloxin [17], a polo-binding box inhibitor (Figure 3C). These results suggested that mutations in the ATP binding pocket of PLK1 abrogated the efficacy of ATP competitive PLK1 inhibitors.

### MDR1 expression was associated with volasertib-resistance

**Figure 4:**
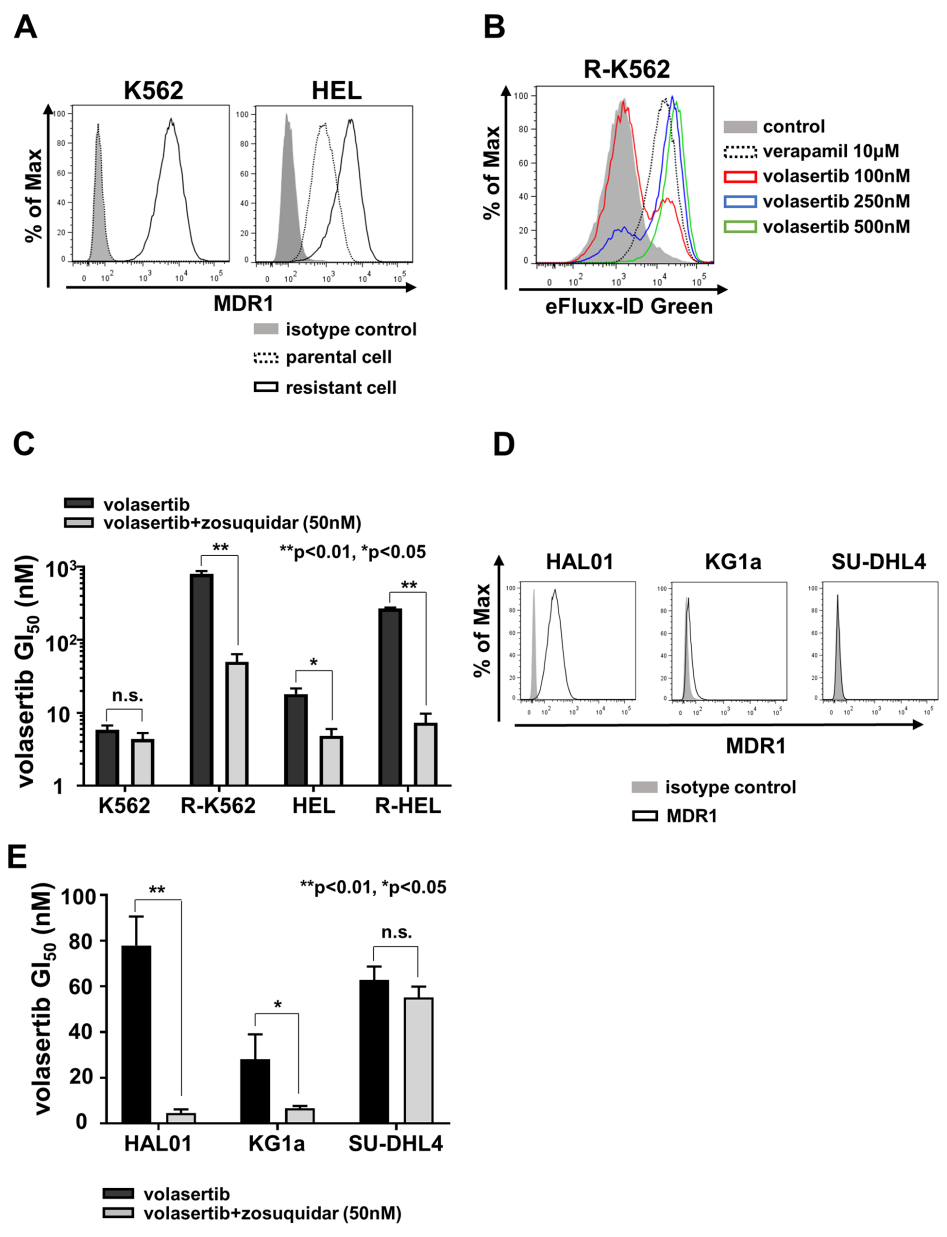
MDR1 expression was associated with volasertib-resistance **(A)** The expressions of MDR1 in both the parental and the volasertib-resistant K562 and HEL cells were assessed by FCM. **(B)** Drug efflux assay was performed in the volasertib-resistant K562 cell using a fluorescent dye which was a substrate of MDR1. **(C)** The GI_50_ values of volasertib in both parental and volasertib-resistant K562 and HEL cells with or without MDR1 inhibitor, zosuquidar. **(D)** The expressions of MDR1 in HAL01, KG1a, and SU-DHL4 cells were analyzed by FCM. **(E)** The GI_50_ values of volasertib in HAL01, KG1a, and SU-DHL4 cells with or without zosuquidar. Error bars represent the mean values ± S.D. of at least three independent experiments.

To clarify the resistance mechanism in *PLK1* non-mutated resistant cells, R-K562 and R-HEL, mRNA and protein expression of multidrug resistance protein 1 (MDR1) were examined by flow cytometer (FCM) (Figure 4A). We identified higher surface expression of MDR1 in R-K562 and R-HEL than in parental cells. In contrast, the *PLK1* mutated volasertib-resistant cell lines, R-MOLM14 and R-HL-60, did not express MDR1 (Supplementary Figure 4A). To determine whether volasertib was excreted through the MDR1, we performed a drug efflux assay using a green fluorescent dye. The exportation of green fluorescent dye in R-K562 cells was dose-dependently inhibited by verapamil and by volasertib (Figure 4B). We next examined the efficacy of the MDR1 inhibitor, zosuquidar [18], in these MDR1 expressing cells. Zosuquidar restored the efficacy of volasertib in these MDR1 expressing volasertib-resistant cells and parental HEL cells, which expressed low levels of MDR1 (Figure 4C). The same effect was also confirmed with verapamil in the volasertib-resistant K562 cells (Supplementary Figure 4B). Taken together, volasertib is a substrate of MDR1 and the higher expression of MDR1 confers resistance to volasertib. Since the GI_50_ values in KG1a, HAL01 and SU-DHL4 cells were relatively higher than in the other cell lines (Figure 1A), we evaluated the expression of MDR1 in these cells and confirmed that HAL01 and KG1a cells expressed MDR1, but SU-DHL4 did not all (Figure 4D). The sensitivity to volasertib in both HAL01 and KG1a cells was restored by zosuquidar (Figure 4E). The MDR1-associated volasertib resistance was also confirmed in the cell lines that had relatively higher GI_50_ values of volasertib.

### The efficacy of combination therapy with volasertib and azacitidine in myeloid leukemia cell lines and primary AML cells

**Figure 5:**
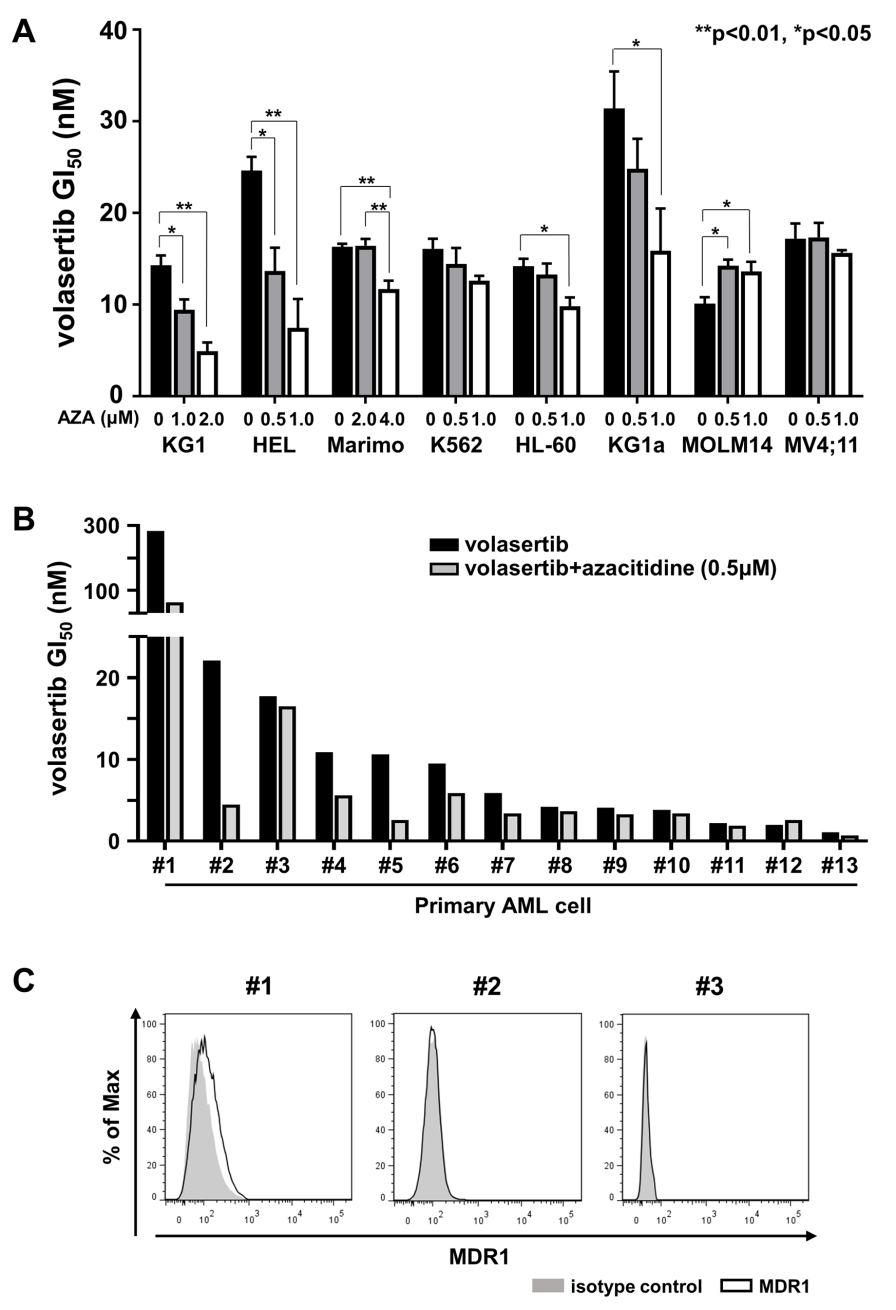
The efficacy of combination therapy with volasertib and azacitidine (AZA) in myeloid leukemia cell lines and primary AML cells **(A)** GI_50_ values of volasertib in volasertib mono-therapy and combination therapy with AZA in myeloid leukemia cell lines. Error bars represent the mean values ± S.D. of at least three independent experiments. **(B)** GI_50_ values of volasertib in mono- and combination-therapy with AZA in primary AML cells. **(C)** The expression levels of MDR1 in primary AML cells.

We examined the combination effects of volasertib and azacitidine (AZA) in the leukemia cell lines. We determined the GI_50_ values of AZA in mono-therapy (Supplementary Figure 5A), and the combination index (CI) of volasertib and AZA. The GI_50_ values of volasertib in KG1, HEL, Marimo, K562, HL-60 and KG1a were lowered when they were co-treated with AZA in a dose dependent manner, whereas combination therapy with AZA did not impact the GI_50_ values of volasertib in MOLM14 or MV4;11 (Figure 5A). Consistent with these results, the CI revealed that the efficacy of AZA was synergic in HEL and KG1, additive in Marimo, K562, HL-60 and KG1a, and antagonistic in MOLM14 and MV4;11 (Supplementary Figure 5B). These results indicated that the combined effects with volasertib and AZA differed among cell lines.

Subsequently, we evaluated the efficacy of volasertib in mono- and combination-therapy with AZA in primary AML cells *in vitro*. Although the efficacy of this combination varied in primary AML cells, it was prominent in the samples with relatively higher GI_50_ values of volasertib in mono-therapy (Figure 5B). We then compared the volasertib GI_50_ value with the expression level of *PLK1* mRNA and cell proliferation rate, but there were no significant correlations between them (Supplementary Figure 5C). We examined the expression levels of MDR1 in three primary AML cells, #1, #2 and #3, which exhibited relatively higher GI_50_ values in volasertib mono-therapy. As weak expression of MDR1 was observed in #1 cells, MDR1 might have caused the resistance to volasertib in this case (Figure 5C).

### Volasertib was more effective in cells in the G2/M phase

**Figure 6:**
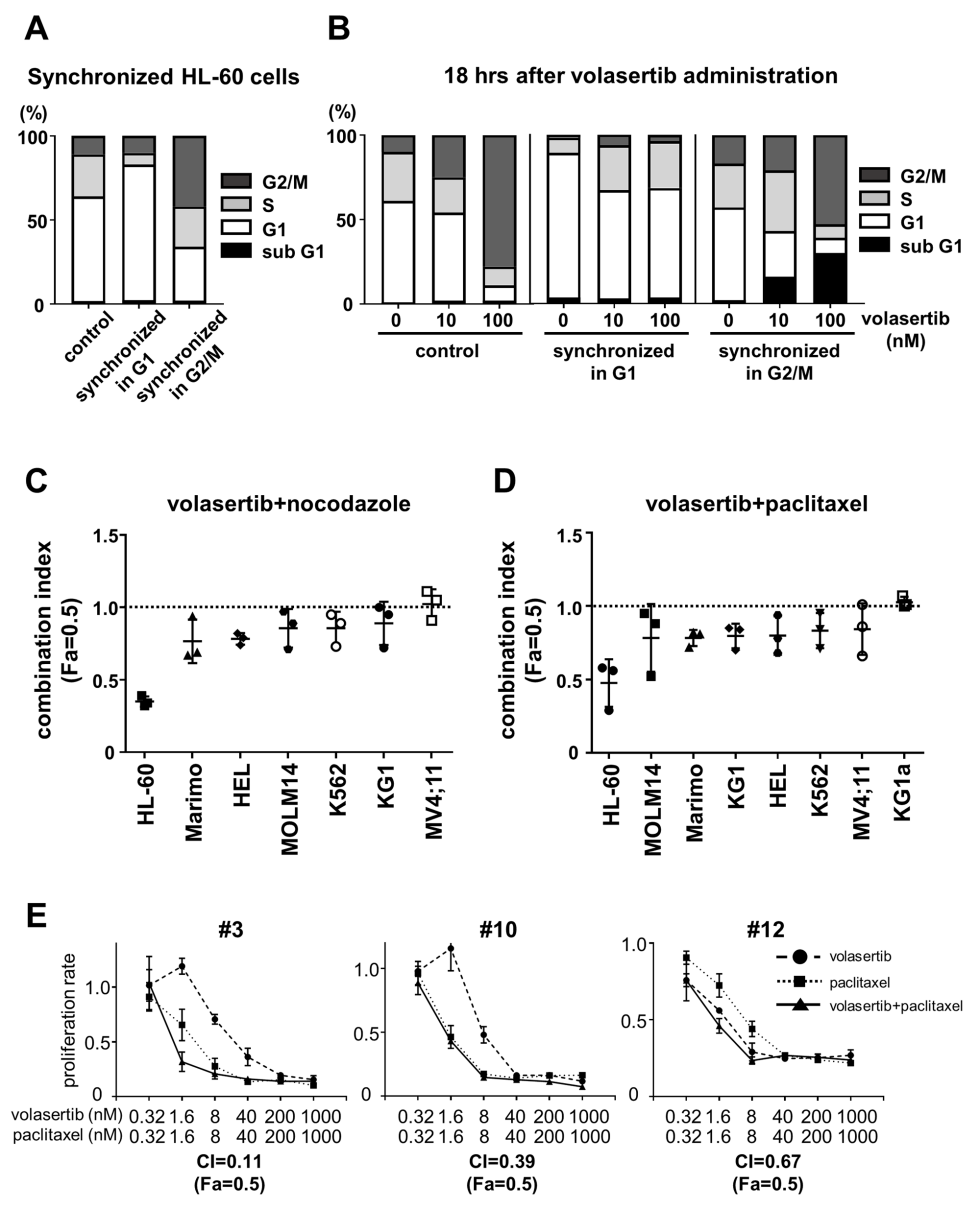
Volasertib was more effective for the cells in G2/M phase than those in G1 phase **(A)** The cell cycle was synchronized in G1 phase and G2/M phase in HL60 cells by single- or double-thymidine block. **(B)** After cell cycle synchronization, HL-60 cells were treated for 18 hours and cell cycle was analyzed by FCM. Bar graphs showed that the frequency of cells per cell cycle. **(C, D, E)** The combination index (Fa=0.5) of combination therapy of volasertib and nocodazole (C), and volasertib and paclitaxel in AML cell lines (D) and in primary AML cells (E). Error bars represent the mean values ± S.D. of at least three independent experiments.

We synchronized the cell cycle in G1 phase and G2/M phase with the thymidine block method (Figure 6A). When the G2/M-arrested HL-60 cells were treated with volasertib, significant G2/M arrest followed by an increase of the subG1 component occurred 18 hours after volasertib treatment, as compared with the control. On the other hand, G2/M arrest followed by the increase of the subG1 population was rarely observed in volasertib treated G1-arrested cells, indicating that the cells in the G2/M phase were much more sensitive to volasertib than those in the G1 phase (Figure 6B). This was confirmed by the synergistic or additive effect of volasertib and nocodazole, an inhibitor of microtubule polymerization, against several kinds of leukemia cell lines (Figure 6C and Supplementary Figure 6A). In addition, the combination effects of volasertib with paclitaxel, which is a microtubule polymer stabilizer, was also effective (Figure 6D and Supplementary Figure 6B). Subsequently, we evaluated combination effects of volasertib and paclitaxel in primary AML cells. The combination therapy exhibits the synergistic effects in primary AML cells as well as cell lines (Figure 6E). These results indicated that the combination therapy of volasertib with the agent that caused cell cycle accumulation in the G2/M phase was effective.

### PI3K inhibitor is a potent combination partner with volasertib

**Figure 7:**
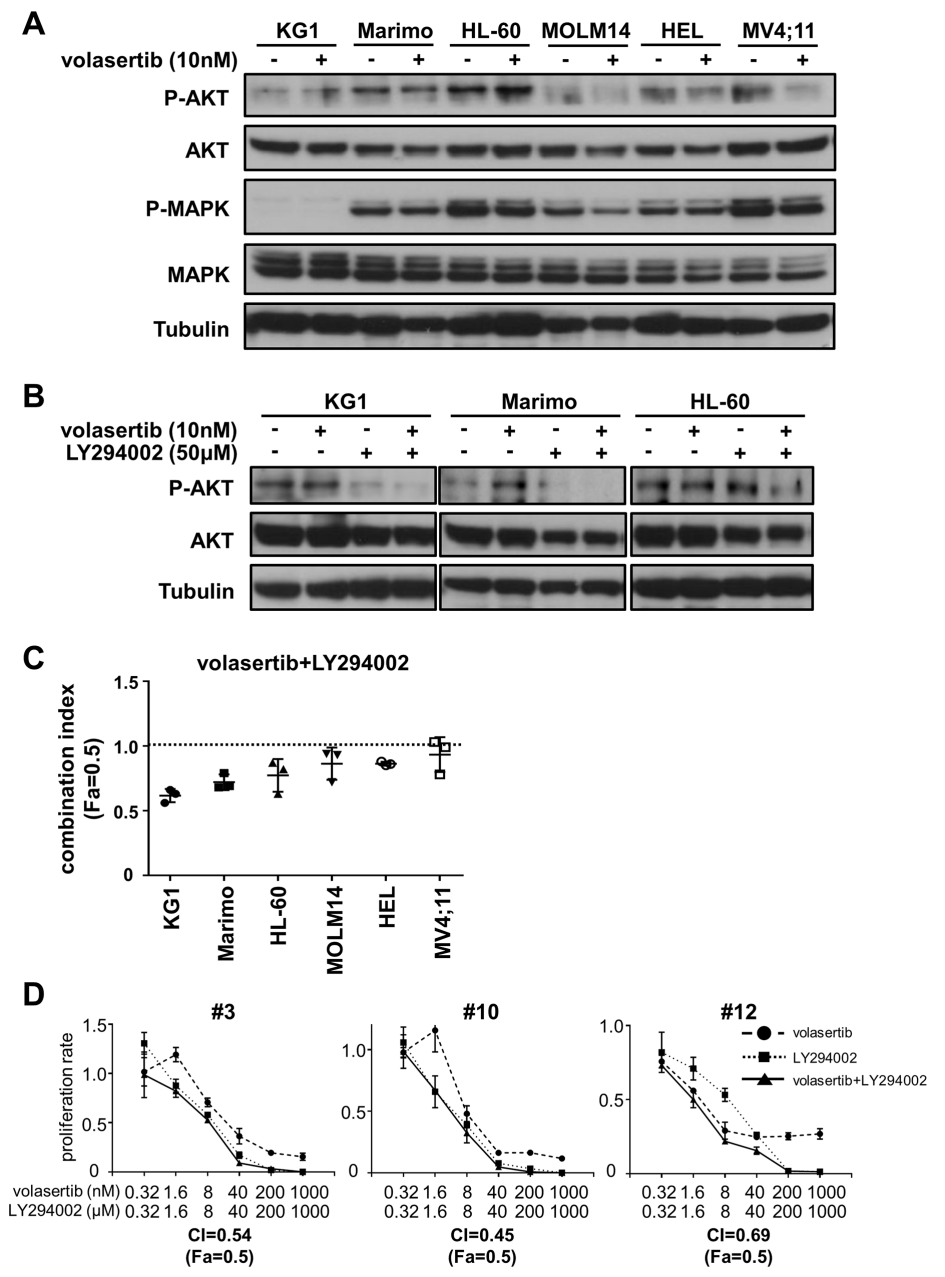
Addition of PI3K inhibitor is a potent combination therapy with volasertib **(A)** Phosphorylation levels of AKT and MAPK in AML cell lines after volasertib administration. **(B)** Phosphorylation levels of AKT in KG1, Marimo and HL-60 cells after the treatment with volasertib and/or LY294002. **(C)** The CI of volasertib and LY294002 treatment in AML cell lines. **(D)** The proliferation rate of primary AML cells with volasertib and/or LY294002 treatment. Error bars represent the mean values ± S.D. of at least three independent experiments.

Since the up-regulation of cell survival signals are closely correlated with drug-resistance, we hypothesized that the signaling pathways involved in cell survival were activated upon volasertib administration. We examined phosphorylation levels of AKT and MAPK after volasertib administration in leukemia cell lines. The phosphorylation levels of AKT in KG1 and HL-60 cells were increased after treatment with volasertib, whereas those in MOLM14, HEL, and MV4;11 cells were decreased (Figure 7A). We next examined the phosphorylation levels of AKT after volasertib and/or LY294002, a potent inhibitor of PI3-kinase/Akt signaling, administration in the cell lines that showed up-regulation of pAKT. The phosphorylation level of AKT was decreased by LY294002 alone and combination with volasertib (Figure 7B). Finally, we studied the combination effects of volasertib with LY294002 in these AML cell lines. The CI indicated that the addition of LY294002 to volasertib provided synergic or additive effects in KG1, Marimo and HL-60 cells (Figure 7C and Supplementary Figure 7). On the other hand, this combination therapy did not exhibit combination effects in cell lines without up-regulation of pAKT upon volasertib administration. We also demonstrated that this combination provides synergistic effects in primary AML cells (Figure 7D). These results indicate that combination therapy with volasertib and PI3K inhibitor is a potent combination therapy against AML, and the pAKT level is a predictive marker for this combination.

## DISCUSSION

We demonstrated high potency of volasertib against leukemia cells; however, the phosphorylation level of PLK1 in the cells without the volasertib treatment was not associated with the sensitivity to volasertib. Since PLK1 is phosphorylated in the G2/M state, an addition of volasertib induces the G2/M arrest, resulting in the increase of the PLK1 phosphorylation level as shown in Figure 1E. On the other hand, volasertib also inhibits the phosphorylation of the PLK1 substrates, such as Wee1 and Cdc25C. This inhibition also induces the G2/M arrest, leading to cell death. Therefore, volasertib-induced cells death might not be associated with the PLK1 phosphorylation levels, and the PLK1 phosphorylation level is not a suitable biomarker for predicting the efficacy of volasertib.

In this study, we investigated the resistant mechanism of volasertib in AML cells using volasertib-resistant cell lines. We identified two resistant mechanisms; one is the acquired mutation in the *PLK1* gene and the other is the overexpression of MDR1. We determined two types of mutations, L59W and F183L, and both amino acid residues were located in the ATP-binding domain. According to the co-crystal structure of PLK1 with BI2536, a predecessor of volasertib with similar chemical structure, L59, which is located in the cyclopentyl of the ATP-binding pocket is thought to be associated with the potency and selectivity of BI2536, and the F183 residue possibly enhances binding affinity through π-π stacking with the pteridinone moiety [19]. Our results proved that the mutations in the ATP-binding domain of PLK1 affected the sensitivity of volasertib in cell-based models and these mutations could have occurred during the volasertib treatment. Moreover, we demonstrated that non-ATP competitive PLK1 inhibitors were still options for PLK-targeting therapy in these volasertib-resistant cells. Therefore, evaluation of these alterations in clinically volasertib-resistant cells is required. It is well known that over-expression of MDR1 preferentially confers resistance to anti-cancer drugs in many cancers including AML [20]. The transportation of anti-cancer drugs by MDR1 was reduced by volasertib because of the G2/M arrest of cancer cells [21]. Furthermore, volasertib inhibited MDR1 ATPase activity in a dose-dependent manner [21]. On the other hand, over-expression of MDR1 conferred resistance to volasertib in cell lines and the sensitivity was restored upon administration of a competitive inhibitor or drug substrate of MDR1 [22]. Our results clearly demonstrated that volasertib was a substrate of MDR1, and the increased expression of MDR1 was associated with the resistance to volasertib in both cell lines and primary AML cells. The combination therapy with MDR1 modulators and/or MDR1 substrate anti-cancer agent is effective to conquer the resistance to volasertib in MDR1-expressing cells.

We examined the combination therapy with volasertib and other agents to optimize the efficacy of volasertib. AZA is widely used for high-risk MDS and elderly AML patients, and the combination therapy of volasertib and AZA has been investigated in clinical trials [23]. We demonstrated that the addition of AZA was effective in most primary AML cells, indicating that the combination therapy of volasertib and AZA is one of the promising therapeutic options for AML patients. However, since the growth inhibitory effects of this combination on AML cells varied, further study is required to identify the predictive factors for this combination therapy. Although cross-resistance between volasertib and AZA has not been fully elucidated, volasertib-resistant cells R-K562 exhibited resistance to AZA as well as volasertib. AZA was not a substrate of MDR1 and the equilibrative nucleoside transporters (hENTs) expression was suggested to be associated with AZA resistance [24]; however, we did not find any difference in hENTs expression between R-K562 and the K562 cells (data not shown).

The protein expression of PLK1 was increased during the G2/M and S phases, and volasertib was more effective against the cells in the G2/M phase than those in the G1 phase. These results suggested that a therapeutic agent that induces cell cycle accumulation in the G2/M phase is a better candidate for combination with volasertib. It was reported that vincristine, an inhibitor of polymerization of microtubules, exhibited synergic effects in combination with volasertib against solid cancer cells [25, 26]. Here, we demonstrated that the other microtubule-targeting agents, nocodazole and paclitaxel, also showed synergistic effects in combination with volasertib. Moreover, it was thought that combination therapy with PLK1 inhibitors potentially reduces or eliminates the paclitaxel resistance in solid cancers [4]. Further study is required to confirm the efficiency and toxicity of these combination therapies for clinical use.

We also demonstrated the efficacy of volasertib in combination with AKT inhibitor, LY294002, for AML cell lines. The PI3K-AKT pathway is vital for cell proliferation and is also associated with the activation of PLK1 in mitotic cells. It has been reported that the phosphorylation of Ser99 in PLK1 by the PI3K/Akt pathway promotes cell mitosis, and inhibition of PI3K/AKT signaling delayed the metaphase to anaphase transition [27]. Thus, the combination of PI3K/AKT inhibitor and PLK inhibitor is thought to be potent in terms of cell cycle regulation. Although this combination effect varied among cell lines in our study, it was concordant with the phosphorylation status of AKT.

In conclusion, volasertib inhibits the proliferation of most leukemia cell lines and primary AML cells *in vitro*. Although PLK1 is over-expressed in a variety of cancer cells, PLK1 is vital for cell proliferation regardless of normal or malignant cells. The combination with more cancer-specific, molecular targeting agents is suitable for the clinical development of PLK1 inhibitors. Further study is required to identify a subset of AML patents with optimal response to volasertib, and the molecules or pathways that associate with the response to volasertib in AML cells.

## MATERIALS AND METHODS

### Reagents

Volasertib, BI2536, rigosertib, paclitaxel, zosuquidar and LY294002 were purchased from Selleck chemicals (Houston, TX), and poloxin was from Sigma-Aldrich (St.Louis, MO).

### Cell lines and cell culture

Human leukemia cell lines; MOLM14 was obtained from Fujisaki Cell Center, Hayashibara Biochemical Laboratories (Okayama,Japan); Kasumi-1 was from Hiroshima University (Hiroshima, Japan); HAL-01 was from Tokyo medical college (Tokyo, Japan) and Marimo, MEG-01 and Sachi were established at Nagoya University. The other cell lines were obtained from the American Type Culture Collection (ATCC, Manassas, VA) or DSMZ (Braunschweig, Germany).

MV4;11 was maintained in Iscove’s Modified Dulbecco’s Medium (IMDM) (Invitrogen, Carlsbad, CA) with 10% fetal calf serum (FCS) (Invitrogen), and the other cell lines were in RPMI1640 medium (Invitrogen) with 10% FCS.

### Establishment of volasertib-resistant cell lines

Volasertib-resistant cell lines (R-MOLM14, R-HL-60, R-MV4;11, R-K562 and R-HEL) were established by culturing parental cells in escalating concentrations of volasertib for several months.

### Patient samples

Bone marrow (BM) mononuclear cells were isolated from BM samples from patients with AML using Ficoll-Paque Plus density gradient centrifugation media (GE Healthcare, Buckinghamshire, UK). Informed consent was obtained from all patients according to the Declaration of Helsinki for banking and molecular analysis. Approval was also obtained from the ethical committees of Nagoya University. Details of patient characteristics are presented in Supplementary Table 1.

### Establishment of mutant PLK1-expressing U937 cells

Human full-length Wt- and Mutant-PLK1 cDNAs were amplified using cell lines and a FLAG-tag sequence was introduced by PCR. These cDNAs were cloned into the pMX-IP vector (kindly provided by Professor Toshio Kitamura, University of Tokyo, Japan) and transduced into U937 as previously described [28, 29].

### Cell growth inhibitory assay

Cells were seeded at 1x10^4^ / well and cultured in 96-well culture plates with or without each inhibitor for three days. Cell viability was determined by the CellTiter96 Proliferation Assay (Promega, Madison, WI). Human primary AML cells were cultured in MethoCult H4534 (StemCell Technologies, Vancouver, Canada) with inhibitors for seven days, and cell viability was measured by the CelltiterGlo Luminescence Cell Viability Assay (Promega). GI_50_ values were calculated using XLfit software (IDBS, Surrey, UK). Combination effects were determined using the combination index (CI), which was calculated by Compusyn software (CompuSyn, Paramus, NJ, USA). A Combination index of less than 0.9 was considered synergistic, from 0.9 to 1.1 was additive and greater than 1.1 was antagonistic.

### Antibodies

The anti-phospho-PLK1 (Thr210) and the mouse anti-PLK1 antibodies were purchased from Abcam (Cambridge, UK). The mouse anti-Wee1, the rabbit anti-phospho-AKT (Ser473), the anti-phospho-p44/42 MAPK (Thr202/Tyr204), anti-AKT and anti-MAPK antibodies were from Cell Signaling Technology (Beverly, MA). The anti-FLAG antibody (clone M2) was from Sigma-Aldrich. Anti-MDR1 conjugated with PE was form Beckman Coulter (Brea, CA).

### Immunoblotting

Cells were lysed with CelLyticM (Sigma-Aldrich) containing protease and phosphatase inhibitors (Sigma-Aldrich). Proteins were separated by SDS-PAGE, and transferred to polyvinyldifluoride (PVDF) membranes (Millipore, Billerica, MA). Membranes were blocked with SuperBlock (TBS) blocking buffer (Thermo Fisher Scientific, Waltham, MA) and incubated with the indicated antibodies. After incubation with anti-mouse or anti-rabbit horseradish peroxidase antibodies (GE Healthcare), ECL Western Blotting Detection Reagents (GE Healthcare) were used to detect the signal.

### Immunofluorescent staining

Cytospine slides were fixed with 4% Paraformaldehyde Phosphate Buffer Solution (Wako Pure Chemical Industries, Japan). After permeabilization with 0.2% Triton X-100 in PBS, the cells were blocked with blocking buffer. Then, they were incubated with primary antibodies, and washed with 0.1% Tween20 (Sigma-Aldrich) in TBS (TBS-T). Subsequently, cells were incubated with secondary antibodies. The cover glasses were mounted on a slide glass with the DAPI/Antifade reagent, Prolong® Gold antifade reagent with DAPI (Invitrogen). Images were acquired from fluorescence microscopes equipped with digital cameras (Axioskop 2, Zuiss) and processed in Axio Vison Rel.4.5.

### Quantitative assessment of PLKs mRNA

Total RNA was extracted using QIAamp RNA blood Mini Kit (QIAGEN, Hilden, Germany) and reverse transcribed using the SuperScript II reverse transcriptase Kit (Thermo Fisher Scientific) according to the manufacturer’s instructions. The expression level of *PLK1*, *PLK2* and *PLK3* transcripts was quantitated using a real-time fluorescence detection method with an ABI prism7300 sequence detection system and Taqman® Gene Expression Assay probe (Applied Biosystems, Foster City, CA). *GAPDH* served as a control for cDNA quality.

### Drug efflux assay

The MDR1-mediated efflux assay was performed using the EFLUXX-ID Green multidrug resistance assay kit (Enzo Life Science, Plymouth Meeting, PA) according to the manufacturer’s instructions. The fluorescence of Green Dye was measured by flow cytometer.

### Cell-cycle analysis and cell cycle synchronization assay

Cell-cycle analysis was performed using propidium iodide (PI) (Sigma-Aldrich) as previously described [30]. To synchronize cells with G1 and G2/M phase, cells were treated with thymidine (Sigma-Aldrich) at a final concentration of 2 mM for 18 hours. Then, cells were washed with PBS and incubated in fresh medium for 10 hours to synchronize cells in the G2/M phase. For G1 phase synchronization, 2 mM of thymidine was added again and cells were incubated for 3 hours.

### Statistical analysis

All statistical analyses were performed with SPSS ver. 25 (IBM, Armonk, NY), and differences with P-values less than 0.05 were considered significant.

## SUPPLEMENTARY MATERIALS FIGURES TABLE


