# Supplementary material for: Identification of volasertib-resistant mechanism and evaluation of combination effects with volasertib and other agents on acute myeloid leukemia
Source: Oncotarget. 2017 Jul 26;8(45):78452–65. doi: 10.18632/oncotarget.19632 (PMC5667974; doi:10.18632/oncotarget.19632)
Supplement: Supplementary file 1 [file oncotarget-08-78452-s001.pdf]

Identification of volasertib-resistant mechanism and evaluation of combination effects with volasertib and other agents on acute myeloid leukemia

SUPPLEMENTARY MATERIALS

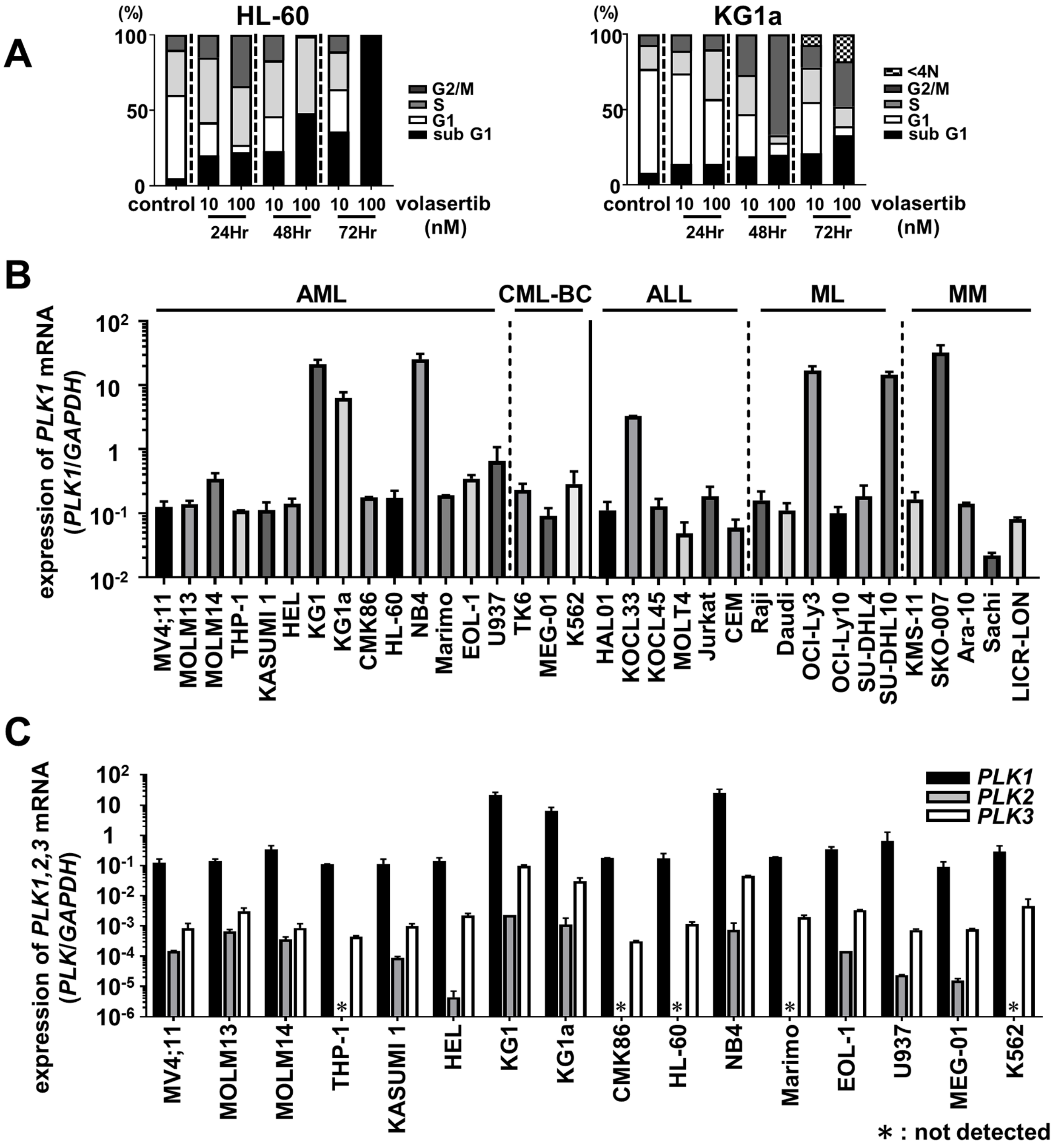

Supplementary Figure 1: **(A)** Bar graphs showed the percentageges of cells in G1, S and G2/M cell cycle at the indicated point after volasertib administration. **(B)** The expression of *PLK1* mRNA in a series of human hematological malignant cell lines. **(C)** The expressions of *PLK1, PLK2,* and *PLK3* mRNA in AML cell lines.

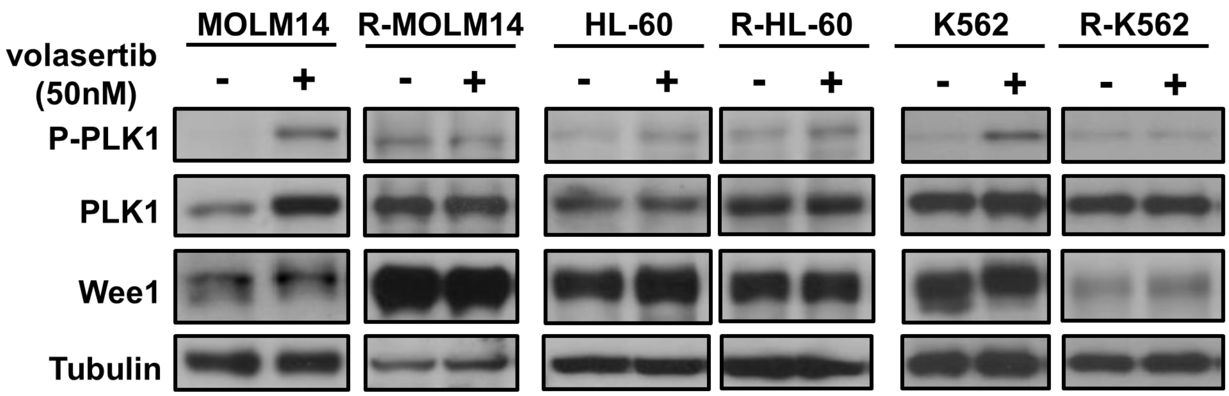

Supplementary Figure 2: The protein expressions of PLK1 and Wee1 were evaluated western blot analysis. Both parental and volasertib-resistant cells were treated with 50 nM volasertib for 18 hours.

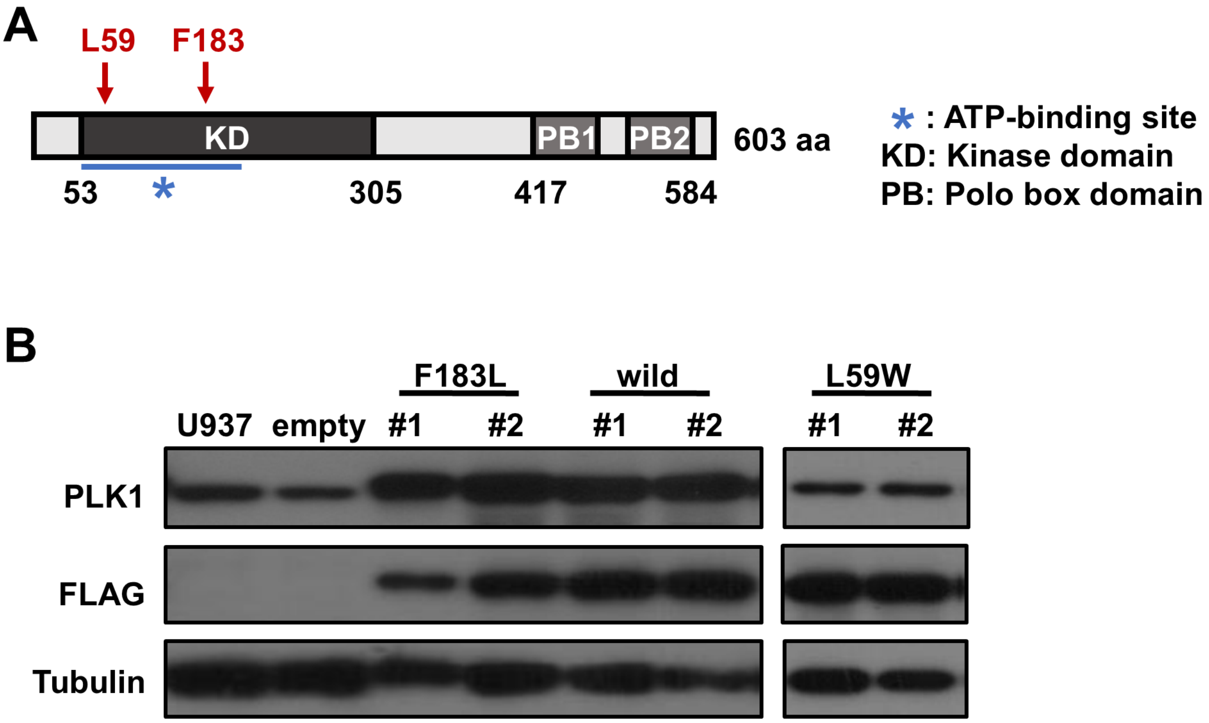

Supplementary Figure 3: (A) The schematic diagram of PLK1 protein. The mutated amino acid residues in volasertib-resistant cells were indicated by red arrows. **(B)** The protein expressions of PLK1 proteins were assessed by western blot analysis with anti-PLK1 and anti-FLAG antibodies.

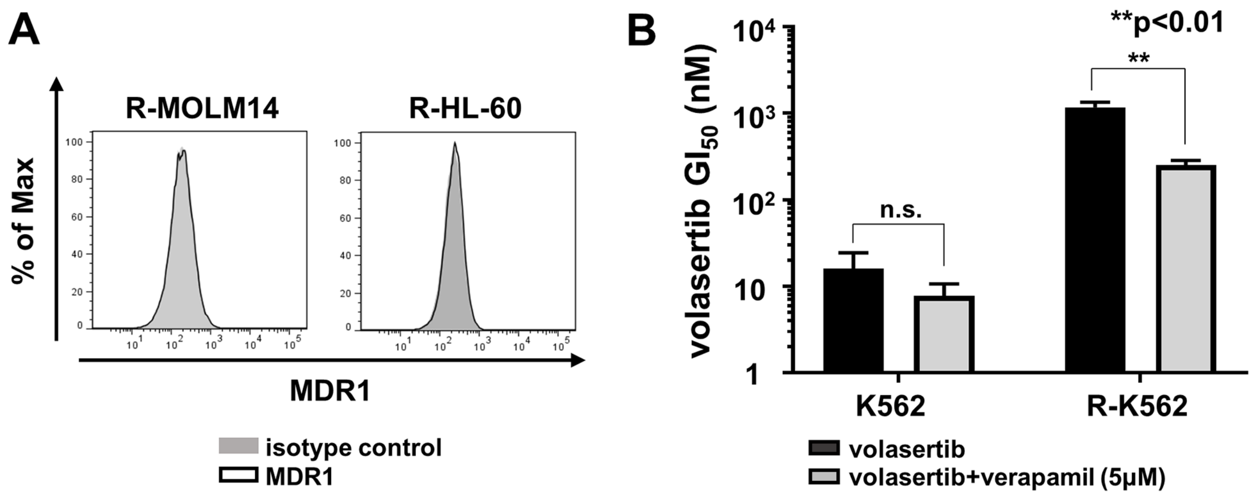

Supplementary Figure 4: (A) The expressions of MDR1 were examined by FCM in volasertib-resistant R-MOLM14 and R-HL-60 cells. **(B)** The GI_{50} values of volasertib in K562 and volasertib-resistant R-K562 cells with or without verapamil. Error bars represent the mean values ± S.D. of at least three independent experiments.

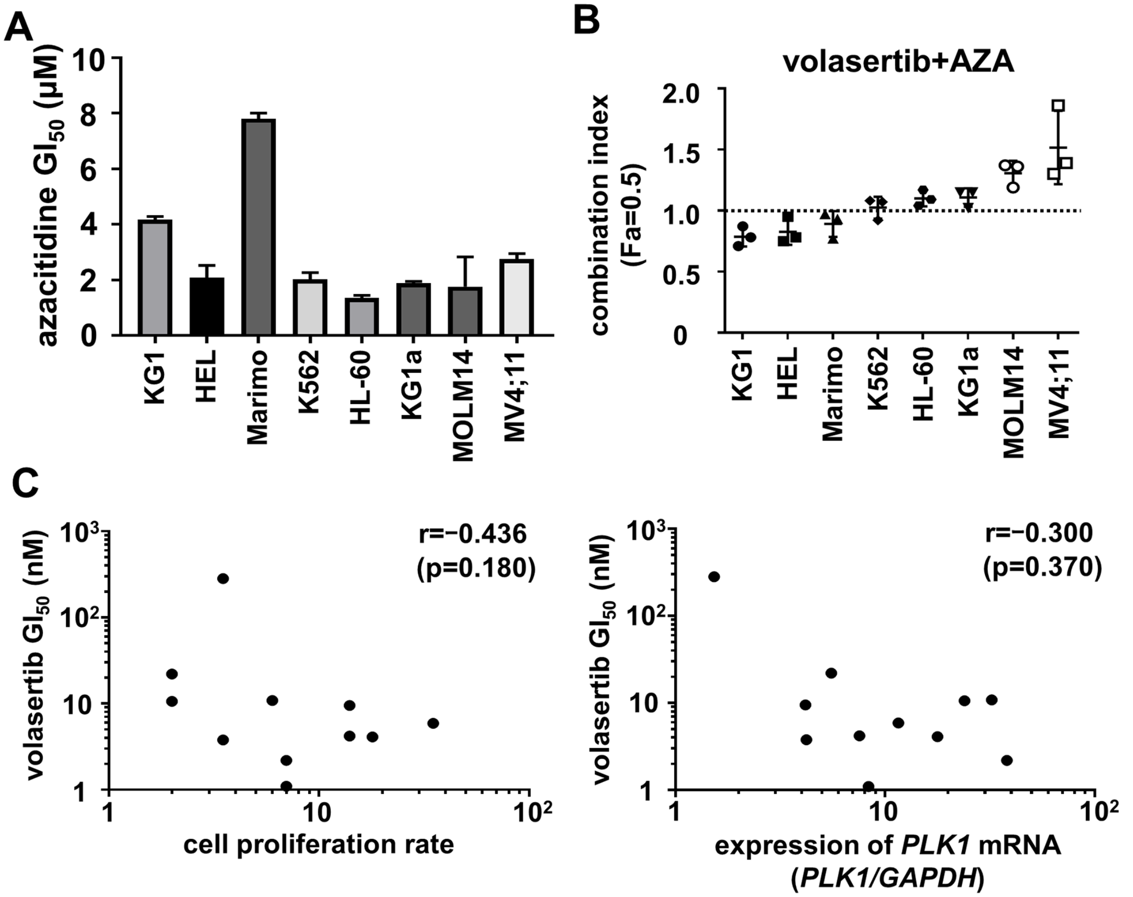

Supplementary Figure 5: (A) GI50 values of AZA in indicated cell lines. **(B)** The combination index (Fa=0.5) of volasertib and AZA was calculated using CompuSyn software. Error bars represent the mean ± S.D. of at least three independent experiments. **(C)** The correlation between the GI_{50} values of volasertib, and cell proliferation rate (left panel) and *PLK1* mRNA expression (right panel) in each AML cells. The correlation coefficient was determined by the Spearman's rank correlation coefficient.

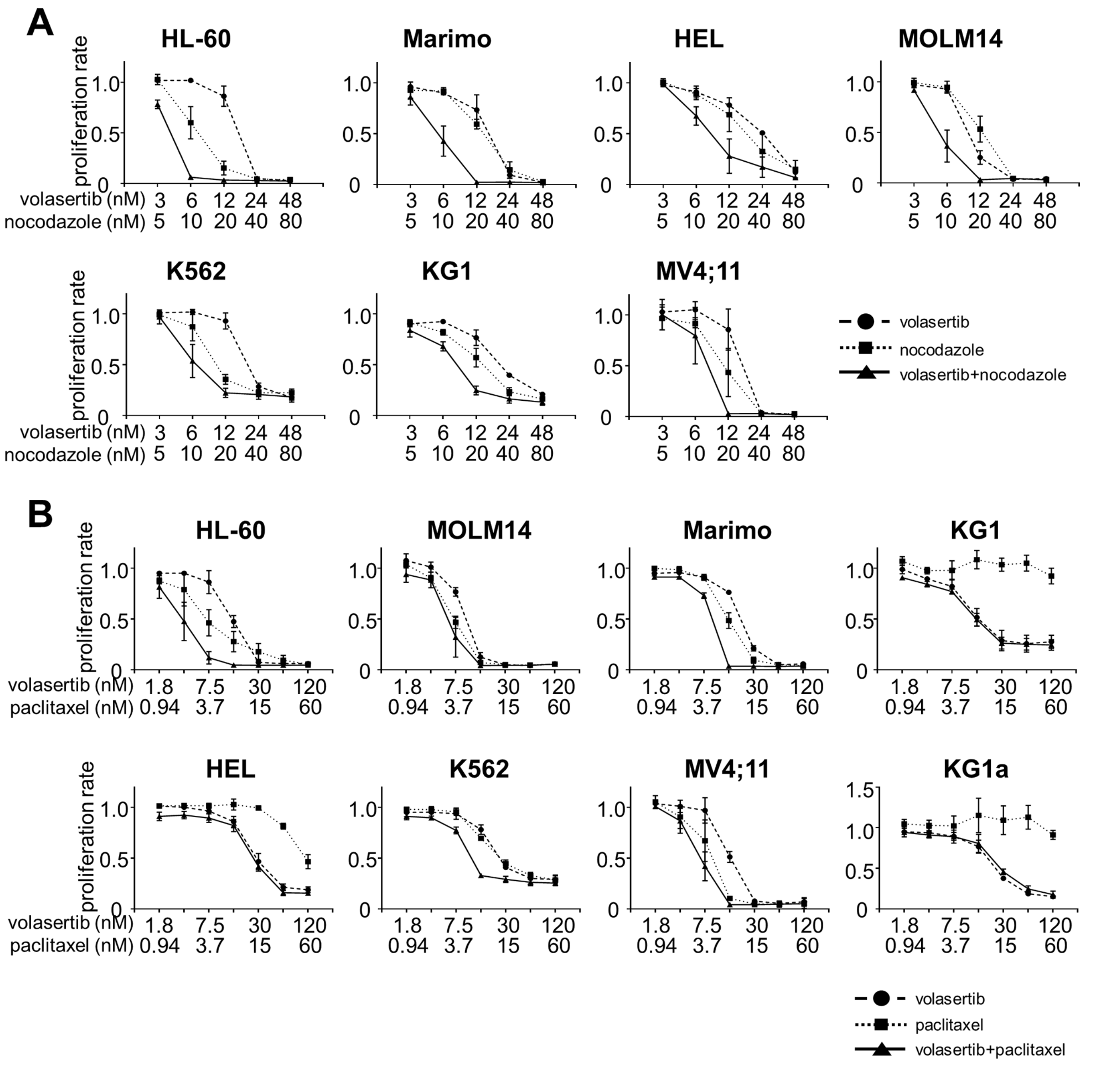

Supplementary Figure 6: The proliferation curves of myeloid leukemia cell lines in the combination with volasertib and nocodazole **(A),** or volasertib and paclitaxel **(B)**. Error bars represent the mean values ± S.D. of at least three independent experiments.

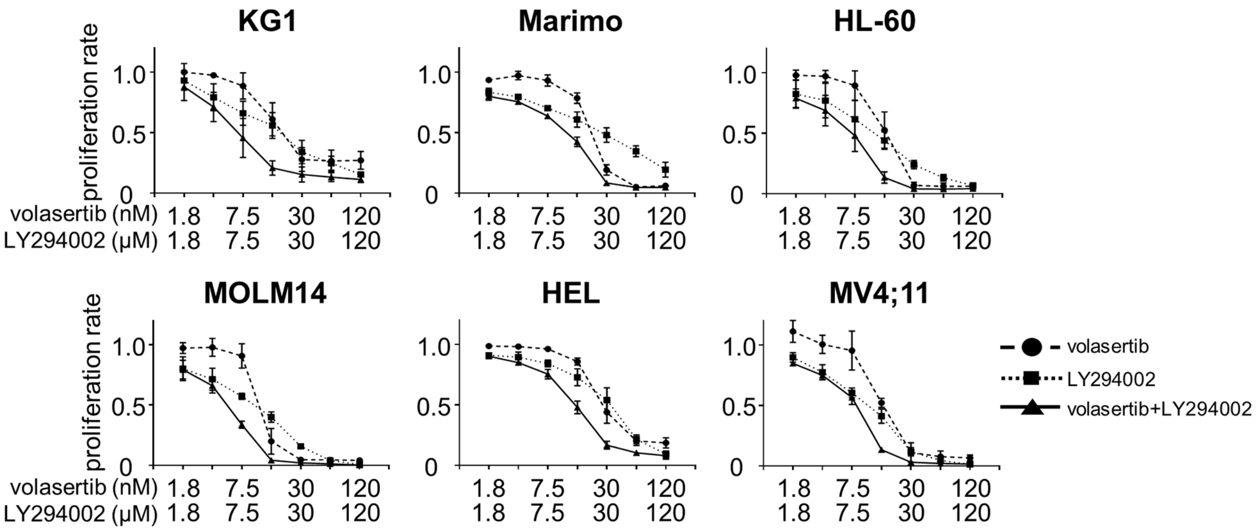

Supplementary Figure 7: The proliferation curves of myeloid leukemia cell lines in the combination with volasertib and LY294002.

Supplementary Table 1: Characteristics of the patient samples in this study

Patient No.	WHO classification	Status	Karyotype	Mutations
#1	AML, NOC	Diagnosis	Normal	
#2	AML, NOC	Diagnosis	Normal	*NPM1, IDH1*
#3	AML with MRC	Relapse	Complex (including -5, -7)	
#4	AML with MRC	Relapse	Complex (including -17)	*TP53, JAK2*
#5	tMN	Diagnosis	Complex (including -5,-7)	
#6	AML, NOC	Diagnosis	Normal	*FLT3*-KDM
#7	AML with MRC	Diagnosis	Normal	*IDH1*
#8	tMN	Relapse	46,XX,t(11;19)(q23;p13;1),i(17)(q10)	
#9	AML, NOC	Diagnosis	Normal	*NRAS*
#10	AML, NOC	Diagnosis	Normal	*FLT3*-KDM
#11	AML with MRC	Relapse	46,X,der(Y)t(Y;1)(q12:q12)	*IDH2*
#12	AML, NOC	Diagnosis	Normal	
#13	AML, NOC	Diagnosis	Complex(including -5, -17)	*IDH2*

NOC, not otherwise categorized; MRC, myelodysplasia related change; tMN, therapy related neoplasm
